# A Jack of All Trades: The Role of Pneumococcal Surface Protein A in the Pathogenesis of *Streptococcus pneumoniae*

**DOI:** 10.3389/fcimb.2022.826264

**Published:** 2022-02-02

**Authors:** Jessica R. Lane, Muralidhar Tata, David E. Briles, Carlos J. Orihuela

**Affiliations:** Department of Microbiology, The University of Alabama at Birmingham, Birmingham, AL, United States

**Keywords:** *Streptococcus pneumoniae*, choline-binding proteins, pneumococcal surface protein A (PspA), pathogenesis, vaccine

## Abstract

*Streptococcus pneumoniae *(*Spn*), or the pneumococcus, is a Gram-positive bacterium that colonizes the upper airway. *Spn* is an opportunistic pathogen capable of life-threatening disease should it become established in the lungs, gain access to the bloodstream, or disseminate to vital organs including the central nervous system. *Spn* is encapsulated, allowing it to avoid phagocytosis, and current preventative measures against infection include polyvalent vaccines composed of capsular polysaccharide corresponding to its most prevalent serotypes. The pneumococcus also has a plethora of surface components that allow the bacteria to adhere to host cells, facilitate the evasion of the immune system, and obtain vital nutrients; one family of these are the choline-binding proteins (CBPs). Pneumococcal surface protein A (PspA) is one of the most abundant CBPs and confers protection against the host by inhibiting recognition by C-reactive protein and neutralizing the antimicrobial peptide lactoferricin. Recently our group has identified two new roles for PspA: binding to dying host cells via host-cell bound glyceraldehyde 3-phosphate dehydrogenase and co-opting of host lactate dehydrogenase to enhance lactate availability. These properties have been shown to influence *Spn* localization and enhance virulence in the lower airway, respectively. Herein, we review the impact of CBPs, and in particular PspA, on pneumococcal pathogenesis. We discuss the potential and limitations of using PspA as a conserved vaccine antigen in a conjugate vaccine formulation. PspA is a vital component of the pneumococcal virulence arsenal – therefore, understanding the molecular aspects of this protein is essential in understanding pneumococcal pathogenesis and utilizing PspA as a target for treating or preventing pneumococcal pneumonia.

## Introduction

*Streptococcus pneumoniae* (*Spn*), or the pneumococcus, is a Gram-positive bacterium that colonizes the nasopharynx. From the nasopharynx, *Spn* can disseminate to normally sterile sites to cause opportunistic infections. These sites include the middle ear, where *Spn* causes otitis media (Bluestone et al., 1992). *Spn *can also be aspirated into the lower respiratory tract to cause pneumonia. These infections typically occur in infants, those who are immunocompromised, or in the elderly (Brooks and Mias, 2018). In approximately 30% of individuals with pneumonia, *Spn* gains access to the bloodstream and the ensuing bacteremia can result in sepsis and disseminated organ damage (Askim et al., 2016; Asner et al., 2019). Within the bloodstream, pneumococci encounter the blood-brain barrier and in rare cases can cross this endothelial cell barrier to cause meningitis (Ring et al., 1998; Thigpen et al., 2011). It is important to consider that *Spn *is primarily an asymptomatic commensal and severe infections are uncommon among otherwise healthy adults. Clinical epidemiological studies show that most affected individuals had at least one or more underlying conditions placing them at higher risk of infection (Bogaert et al., 2004). Pneumococcal infections, even among those who are susceptible, are commonly preceded by prior viral exposure (O'Brien et al., 2000; Jansen et al., 2008). Viral infections, particularly influenza, have been shown to prime mucosal epithelial cells for bacterial binding, enhance carbohydrate and protein levels in airway secretions, and disarm or deflect the immune system. These events enhance the susceptibility of the lower airway for bacterial establishment and, once pneumonia develops, can accelerate the progression towards invasive disease (Hament et al., 1999; Diavatopoulos et al., 2010; Loughran et al., 2019; Van Der Poll and Opal, 2009). 

In 2018, the Centers for Disease Control and Prevention reported that pneumococcal pneumonia resulted in 150,000 individuals being hospitalized in the United States, while pneumococcal bacteremia and meningitis caused 3,500 deaths (Centers for Disease Control and Prevention, 2018). Across the globe, pneumococcal burden was estimated to be 26.7 incidences per 1,000 people causing over one million deaths (Global Burden of Disease Lower Respiratory Infections Collaborators, 2018). Multiple vaccines are currently licensed to protect against *Spn *disease and all are composed of capsular polysaccharide from *Spn*’s most prevalent serotypes (Scott et al., 2021). Since the introduction of a 7-valent conjugate vaccine in 2000, followed by the 13-valent version in 2010, invasive pneumococcal disease (IPD) in children <5 years has drastically decreased by as much 93% in the United States (Centers for Disease Control and Prevention, 2018). In adults, there has been an overall reduction of IPD incidence from 16 to 8 cases per 100,000 (Centers for Disease Control and Prevention, 2018). However, the threat of vaccine escape, serotype 3 for which the vaccine has poor efficacy, and remnant disease caused by non-vaccine serotypes, has kept efforts to improve on these vaccines a top priority (Scott et al., 2021). Along such lines and in 2021, 15- and 20-valent conjugate vaccines were approved, and these will most likely further lower pneumonia rates in the near future (Hurley et al., 2020). Despite the tremendous success of the conjugate vaccines, *Spn* remains a leading cause of community-acquired pneumonia and invasive disease (Global Burden of Disease Lower Respiratory Infections Collaborators, 2018).

*Spn* has several virulence factors that aid its survival within the host, one of these being its capsular polysaccharide which protects it from phagocytosis by immune cells (Hyams et al., 2010). Another being the toxin pneumolysin, which forms pores in the membranes of host cells resulting in ion dysregulation and, at higher concentrations, cell death by apoptosis or necroptosis (Hirst et al., 2004; González-Juarbe et al., 2015). Pneumococcal pneumonia is characterized by a strong inflammatory response in the airway that results in lung consolidation and loss of gas exchange. Pneumolysin, in addition to killing cells and causing the release of alarmins, activates the classical complement cascade (Mitchell and Dalziel, 2014). Lipoteichoic (LTA) and cell wall teichoic acid (WTA) associated with the pneumococcus are Toll-like receptor 1/2 ligands and therefore are also inflammatory (Draing et al., 2006). Phosphorylcholine (PC) residues that are present on LTA and WTA mimic the molecular structure of host platelet-activating factor and bind to platelet-activating factor receptor (PAFr) (Cundell et al., 1995). This activates host cells, resulting in chemokine production, and the PC residues on the surface of the bacterium are targeted by C-reactive protein, which activates complement and exacerbates inflammation (Pepys and Hirschfield, 2003). The pneumococcus is generally protected by its capsule from killing by infiltrated immune cells until the host develops capsule-specific antibody, which then effectively opsonizes the bacterium for phagocytosis.

Clinical isolates of *Spn* vary considerably in their genetic content, as much as 10-15% between strains, and carry between 10-16 choline-binding proteins (CBP) (Hiller et al., 2007; Gisch et al., 2013). With exception to serotype 1 (Cornick et al., 2017), the majority of *Spn* produce pneumococcal surface protein A (PspA), a 65 to 99-kDa CBP that protects the bacteria from C-reactive protein-mediated activation of complement and from killing by lactoferricin, a cationic antimicrobial peptide (Hammerschmidt et al., 1999; Tu et al., 1999). Recent findings from our group describe two new functions for PspA, as an adhesin and means to co-opt host metabolic enzymes for its benefit (Park et al., 2021a; Park et al., 2021b). Indeed, this protein acts as a “Jack of All Trades.” In this review, we will summarize how PC and CBPs contribute to pneumococcal pathogenesis and the role PspA plays during infection. At conclusion, we will discuss how our new understanding of PspA virulence provides insight into *Spn* pathogenesis and the implications towards new treatments and potentially improved vaccines. 

### Phosphorylcholine on the Pneumococcal Surface

Pneumococcal cell wall is located outside the cell membrane and underneath the capsular polysaccharide layer of *Spn*. It is composed of peptidoglycan chains cross-linked to each other with interlaced teichoic acid (Galán-Bartual et al., 2015). The cell wall also acts as an anchor point for capsule types that rely on the Wxy/Wzy-dependent synthesis pathway (Geno et al., 2015). PC is a small amino alcohol that is essential for eukaryotic cell growth but also commonly found on the surface of bacterial pathogens (García et al., 1998; Harnett and Harnett, 1999). Choline is not synthesized by *Spn*, yet the bacteria is dependent on it for growth or one of its structural analogues, such as ethanolamine (Galán-Bartual et al., 2015). The *lic* operon in *Spn* encodes the transporters and enzymes necessary for the uptake and conversion of environmental choline into PC, eventually incorporating it into either LTA or WTA (Zhang et al., 1999). 

LTA and WTA are composed of four to eight repeating units of ribitol 5-phosphate, *N*-acetyl-D-galactosaminyl (GalNAc), 2-acetamido-4-amino-2,4,6-trideoxy-D-galactose (AATGal), and D-glucose (Behr et al., 1992; Jennings et al., 1980). The number of PC residues on the pneumococcal surface varies between strains and can also be influenced by phase-variation (Weiser et al., 1994; Kim and Weiser, 1998). PC on the surface of the pneumococcus functions as a mimetic of platelet-activating factor and thereby binds PAFr on host cells (Cundell et al., 1995; Ring et al., 1998). Its binding by pneumococcal PC results in MAPK activation as well as recruitment of β-arrestin and clathrin to the receptor base for its internalization (Ishii and Shimizu, 2000; Radin et al., 2005; Stafforini, 2009; Asmat et al., 2014). Subsequently, clathrin-mediated endocytosis occurs with uptake of the receptor-bound bacteria into an endosome. Thus, PC plays a vital role in pneumococcal adhesion and the invasion of non-immune cells. Its lowered expression has been shown to result in decreased levels of colonization of the upper airway in a murine model of infection (Kharat and Tomasz, 2006). 

PC on the surface of microorganisms is an important target for the host response. C-reactive protein (CRP), which is made by the liver in response to IL-6, binds to PC activating the complement cascade (Volanakis and Kaplan, 1971; Thompson et al., 1999). Classical complement proteins such as C1q, C4, and C2 recognize and bind to PC-bound CRP with subsequent activation of the cascade and opsonization of the bacterial surface (Rupprecht et al., 2007). Additionally, an anti-phosphorylcholine (anti-PC) IgM autoantibody is produced by CD5^+^ B-cells that provides another layer of host defense against *Spn* or other PC-bearing pathogens (Briles et al., 1981). Notably, anti-PC antibodies are ubiquitous in human sera with up to 10% of total IgM being reactive to PC (Su et al., 2006; Chou et al., 2008). 

## Choline-Binding Proteins

CBPs generally consist of three major domains: a leader peptide, a variable biologically functional domain sometimes followed by a proline-rich domain (PRD), and a conserved choline-binding domain (Bergmann and Hammerschmidt, 2006). The majority of CBPs contain a choline-binding domain at the C-terminal end with some exceptions such as LytB and LytC, where the domain is present at the N-terminus, while for CbpL it is present in the central region of the protein (Bergmann and Hammerschmidt, 2006). The choline-binding domain is modular with a variable number of repeating units consisting of a ~20 amino acid-sequence, i.e., choline-binding motifs. Only four to five choline-binding motifs are required for interaction with PC on LTA or WTA to occur and for the CBP to become docked onto the pneumococcal surface (Yother and White, 1994). The majority of *Spn* strains carry two PC residues per repeating unit of AATGal and GalNAc on LTA or WTA and these are linked *via* a phosphodiester bond to the O6 molecule of GalNAc (Vollmer et al., 2019). Choline-binding motifs are spaced so that the choline-binding domain becomes interlaced with the repeating PC residues.

**Figure 1:**
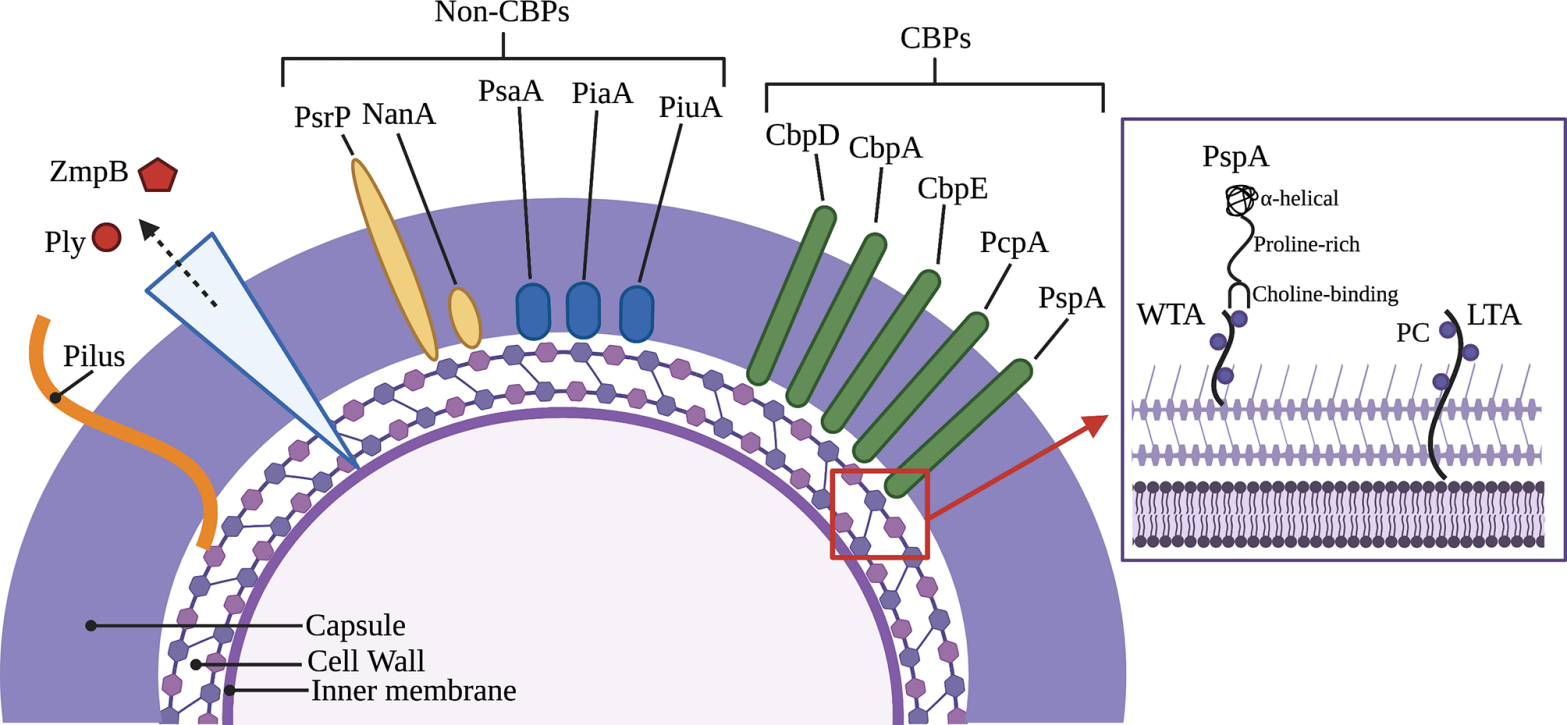
Major virulence factors of *Streptococcus pneumoniae* including choline-binding proteins. Inset picture shows PspA bound to phosphorylcholine on wall teichoic acid and lipoteichoic acid as part of the cell wall and membrane, respectively. Key (left to right): Pneumolysin (Ply), Immunoglobulin A1 protease (ZmpB), Pneumococcal serine-rich repeat protein (PsrP), Neuraminidase (NanA), Pneumococcal surface adhesin A (PsaA), Pneumococcal iron acquisition A (PiaA), Pneumococcal iron uptake A (PiuA), Choline-binding protein D (CbpD), Choline-binding protein A (CbpA), Choline-binding protein E (CbpE), Pneumococcal choline-binding protein A (PcpA), Pneumococcal surface protein A (PspA), Wall teichoic acid (WTA), Phosphorylcholine (PC), Lipoteichoic acid (LTA).

Most strains of *Spn* encode more than 10 CBPs in their genome (Hakenbeck et al., 2009). Among those best characterized are the cell wall hydrolytic enzymes LytA, LytB, and LytC, the pneumococcal surface protein A (PspA), pneumococcal choline-binding protein A (PcpA), and the choline-binding proteins CbpA and CbpC (**Figure 1**) (Hakenbeck et al., 2009). For a complete list of pneumococcal CBPs and their function during colonization and pathogenesis see **Table 1**. Unfortunately, the nomenclature of CBPs becomes complicated as proteins have more than one name and related proteins have similar names and abbreviations. For instance, choline-binding protein A (CbpA) and pneumococcal surface protein C (PspC) are the same protein (Rosenow et al., 1997; Gosink et al., 2000). Herein, we will discuss PspA and CbpA, and will use these names to maintain distinction. 

**Table 1:** Choline-binding proteins of *Streptococcus pneumoniae*.

Name	Abbreviation	Function
Autolysin A	LytA	*N*-acetylmuramoyl-L-alanine amidase; fratricide; caspsule shedding; lysis-mediated release of pneumolysin (Tomasz and Westphal, 1971; Mitchell et al., 1997; Kietzman et al., 2016)
Autolysin B	LytB	Separation of daughter cells via *N*-acetylglucosamine (García et al., 1999; Gosink et al., 2000)
Autolysin C	LytC	Lysozyme; fratricide; binds extracellular DNA to facilitate biofilm formation (Gosink et al., 2000; Eldholm et al., 2009)
Choline-binding protein A	CbpA (also PspC)	Binds laminin receptor; binds polymeric immunoglobulin receptor; mediates bacterial uptake and translocation across epithelial and endothelial layers; binds C3, binds serum factor H (Zhang et al., 2000; Duthy et al., 2002; Orihuela et al., 2004b; Orihuela et al., 2009; Brown et al., 2014)
Choline-binding protein D	CbpD	Competence-mediated fratricide (Gosink et al., 2000; Eldholm et al., 2009)
Choline-binding protein E (also phosphorylcholine esterase)	CbpE (also Pce)	Curates PC residues on pneumococcal surface (Gosink et al., 2000; Hermoso et al., 2005)
Choline-binding protein F	CbpF	Immunity protein protects against autolysis by LytC (Molina et al., 2009)
Choline-binding protein G	CbpG	Serine protease; adhesin (Gosink et al., 2000; Mann et al., 2006)
Choline-binding protein I	CbpI	Adhesin; immune evasion (García et al., 1988; Frolet et al., 2010)
Choline-binding protein J	CbpJ	Adhesin; immune evasion (Frolet et al., 2010; Yamaguchi et al., 2019)
Choline-binding protein K	CbpK	Adhesin (Gosink et al., 2000; Tettelin et al., 2001)
Choline-binding protein L	CbpL	Invasion; immune evasion (Frolet et al., 2010; Gutiérrez-Fernández et al., 2016)
Choline-binding protein M	CbpM	Adhesin; immune evasion (Frolet et al., 2010; Afshar et al., 2016)
Pneumococcal surface protein A	PspA	Blocks C-reactive protein; binds lactoferricin; binds GAPDH; binds LDH (McDaniel et al., 1984; McDaniel et al., 1986; McDaniel et al., 1987; Shaper et al., 2004; Mukerji et al., 2012; Park et al., 2021a; Park et al., 2021b)
Pneumococcal choline-binding protein A	PcpA	Adhesin; aggregation (Sánchez-Beato et al., 1998)

### Structure and Variability of PspA

**Figure 2:**
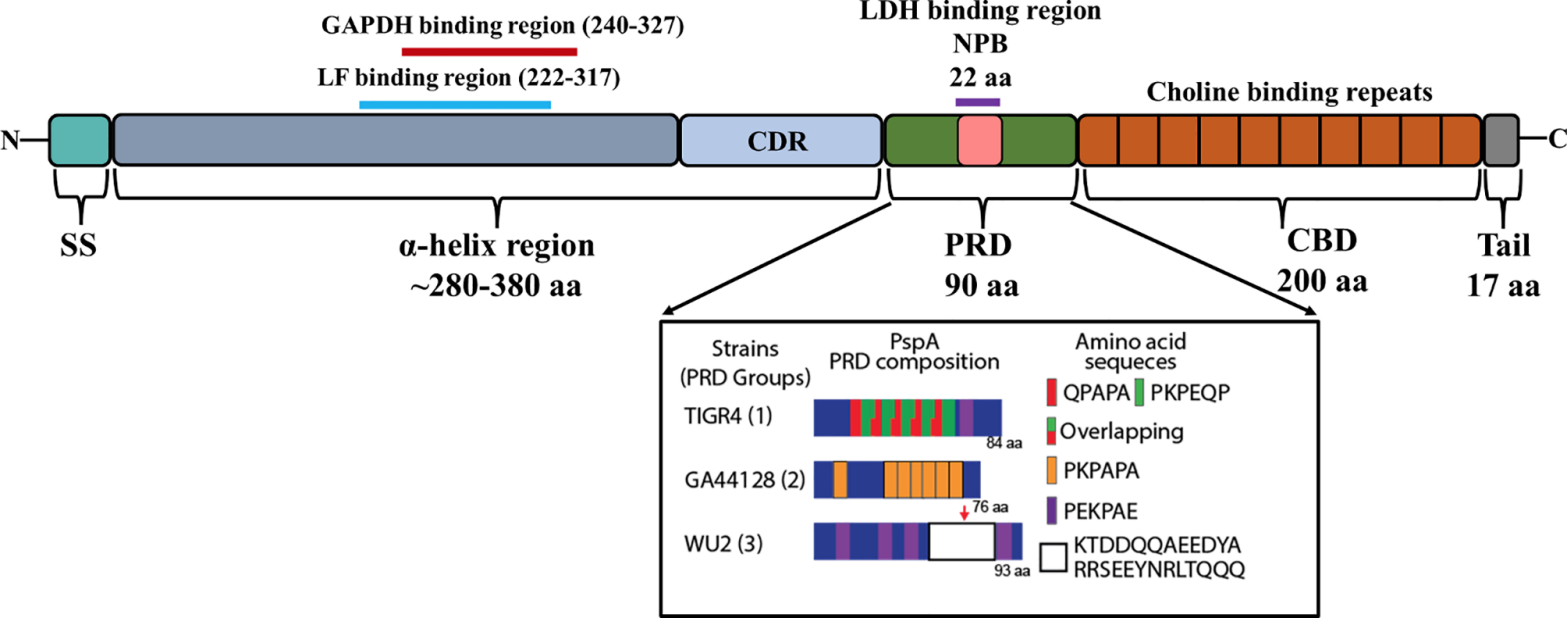
Domain structure of PspA. Illustration shows the N-terminal signal sequence (SS), 280–380 aa α-helix region (αHD), the ~90 amino acid proline rich domain (PRD), and the ~200 aa choline binding repeat domain (CBD) with its short 17 aa C-terminal tail. The clade-defining region (CDR) within the αHD is represented as light blue. The GAPDH binding region (240-327) and Lactoferrin (LF) binding region (222-317) within αHD of WU2 serotype 3 are represented as red and cyan lines, respectively. The non-proline block (NPB)/Lactate dehydrogenase (LDH) binding region within PRD domain is boxed as pink (based upon Mukerji et al., 2018). The PRD composition of PspA from TIGR4, GA44128 and WU2 strains representing each group of PRD are shown in inset (adapted from Park et al., 2021a).

PspA is an abundant surface-exposed virulence factor. It is found in nearly all clinical isolates of *Spn*, but not in other Streptococci (Crain et al., 1990). Transcriptomic studies suggest it is expressed across all anatomical host sites and organs during infection (D'Mello et al., 2020). Based on the sequence variations among strains and differences in reactivity with antibodies, it is now recognized that PspA proteins are mosaics. The size of mature PspA ranges from 65 to 99 kDa across various strains consisting of the three major domains: the N-terminal alpha-helical, the proline-rich, and the C-terminal choline-binding (**Figure 2**). 

The N-terminal region of PspA is 280-380 amino acids (aa) long, immunogenic, and highly variable. It is composed of an antiparallel coiled-coil alpha-helical charged domain (αHD) in an elongated rod-like shape. The charge on the αHD is significantly polarized being electropositive at the C-terminal end and electronegative at the N-terminal end (Lamani et al., 2000). This polarization helps the interaction between PspA and the negatively charged capsule while also decreasing the antibacterial phagocytic activity (Nomura and Nagayama, 1995). The C-terminal 100 aa’s of αHD consist of a clade-defining region (CDR) which is the basis for the classification of PspA into 3 families (Hollingshead et al., 2000). Each family is divided into clades, with Family 1 comprised of clade 1 and 2; Family 2 comprised of clades 3, 4 and 5; and Family 3 comprised of the rarer clade 6. Primary amino acid sequences within the same clade exhibit ≥90% identity compared to only ≤55% identity across different families. The first 100 N-terminal aa’s and the last 100 C-terminal aa’s of the αHD can also elicit a protective antibody response against *Spn* (McDaniel et al., 1994; Roche et al., 2003; Vadesilho et al., 2014) and for this reason are thought to be highly variable between strains. Even though there is significant cross-reactivity of antibodies against different families of PspA, protection is not always guaranteed across different families, an important consideration for its inclusion in any future vaccine formulations. 

The PRD domain consists of repeats of a 6-7 aa motif and can occur several times in any sequence with their diversity well-characterized by Mukerji et al. (2018). Based on the primary amino acid sequence of PspA in 123 pneumococcal isolates, PRD has been divided into three distinct groups. A common motif that is present in many PRD is PAPAPAPA; in some cases, this motif is truncated or interrupted by other amino acids or overlap with other motifs. Another short amino acid motif present in PRD sequences is PKPEQP, occurring in 96% of Group 1 and 73% of Group 3 strains, while it is absent in Group 2 (Mukerji et al., 2018). There are also other differences in motif patterns, for example, in Group 3 the repetition of motifs occurs less frequently and are more dispersed across the PRD, while in Group 1 only a few of the motifs were repetitive, QPAPA or PAPA. Another distinguishing feature of Group 3 PRD-domains in relation to other groups is the presence of a 22 aa non-proline block (NPB) “QQAEEDYARRSEEEYNRLTQQQ”. The NPB is highly conserved, however, a significant single amino acid polymorphism was observed within the NPB immediately preceding ‘‘QQQ”. There were other significant variations observed in the flanking regions of NPB (Mukerji et al., 2018), however the biological significance of these variations is unknown. Both the NPB and the PKPEQP motifs of PRD elicited an immune response and provided protection in mice from a fatal pneumococcal infection caused by isolates that carried a version of PspA with these motifs (Daniels et al., 2010). Notably, the PRD motif is not exclusive to PspA and is also found in CbpA (Rosenow et al., 1997). CbpA, like PspA, has N-terminal alpha-helical domains with the PRD motif positioned between these and the CBD (Luo et al., 2005). 

Due to its surface exposure and the fact that antibodies against PspA help eradicate the bacteria, PspA is under considerable immunological pressure. Accordingly, the presence of mosaic gene structure and diversity in PspA sequences across various *Spn* are indicative of intraspecies horizontal gene transfer and genetic recombination. These derivatives, in turn, are positively selected within the host as they are a means to evade the adaptive immune response against PspA. Notably, the conserved nature of PspA’s repeat motifs imply a direction of evolution where these motifs not only provide antigenic variability, but also confer functions to PspA that impact its virulence. One clear example of this is the NPB (detailed below).

## Established Roles for PspA in Pneumococcal Virulence

PspA was discovered by McDaniel et al. as a result of studies that identified a monoclonal antibody against *Spn* that was not specific against its capsule but was protective against bacterial challenge (McDaniel et al., 1984). The antigen was subsequently recognized as being a surface protein, opening the possibility of protein-based vaccines against the pneumococcus. Subsequent studies by McDaniel and others showed that PspA was required for pneumococcal virulence and that PspA was a CBP. Further studies over the next decade by McDaniel along with Briles and Yother introduced details about the complexity of PspA and established its critical role in pneumococcal pathogenesis and virulence (McDaniel et al., 1987; Briles et al., 1988; Crain et al., 1990; Yother and Briles, 1992; Yother and White, 1994; Briles et al., 1996).

### PspA Regulation

The gene encoding PspA is in the chromosome and not part of a polycistronic operon. Upstream of *pspA* in strain TIGR4 is a 289 bp intergenic region that presumably regulates its expression (Tettelin et al., 2001; Ribeiro et al., 2012). Two-component signal transduction systems (TCSTS) function to sense environmental cues and regulate pneumococcal gene expression (Gómez-Mejia et al., 2018). It has been reported that *pspA* expression is positively regulated *via* the VicRK TCSTS and that the phosphorylated VicR (YycF) response regulator increases its binding upstream of the gene (Ng et al., 2005). The binding of VicR-P protected these regions from digestion by DNase I, suggesting enhanced transcription regulation of PspA (Ng et al., 2005). Additionally, there is a second TCSTS in *Spn* that regulates PspA called RR/HK06, which also regulates CbpA (Standish et al., 2007). *pspA* expression was repressed when the response regulator RR06 was phosphorylated and overexpressed (Standish et al., 2007). A recent report by Im *et al.* also showed *pspA* gene expression is influenced by carbon-catabolite repression and nutrient availability (Im et al., 2021). Altogether, one can infer that the regulation of *pspA* is multifaceted and complex.

Importantly, there have been numerous reports that show *pspA* expression is responsive to the host environment (Gupta et al., 2009; Ogunniyi et al., 2002; Orihuela et al., 2004a; Lemessurier et al., 2006). Current efforts to characterize *in vivo* PspA gene expression include dual species RNA-seq of infected organs from mice using multiple bacterial strains (D'Mello et al., 2020). Using *Spn* serotypes 2, 4, and 6A, our group found that *pspA* was one of the 100 most highly expressed genes overall and that this high level of expression was shared across all anatomical sites of infection including the nasopharynx, lungs, blood, heart, and kidneys (D'Mello et al., 2020). Viral infections can enhance dispersal of pneumococci from biofilms under different conditions, such as during a fever. Interestingly, increased expression of *pspA* was observed when pneumococci were exposed to influenza A virus (IAV). This occurred when pneumococci were grown either planktonically or as a biofilm (Pettigrew et al., 2014). Expression of *pspA* was also observed to be increased in pneumococci that had recently dispersed from a biofilm versus those growing as a biofilm (Pettigrew et al., 2014).

### Complement Evasion

Complement is an enzymatic, self-amplifying cascade that involves the deposition of opsonizing proteins on the surface of bacteria that facilitate their recognition and uptake by phagocytes along with the release of chemotactic and activating factors that attract and recruit immune cells to the site of infection. Complement culminates in the formation of the membrane-attack complex (MAC) which has bactericidal effects, although the thick Gram-positive cell wall of the pneumococcus is resistant to this process (Loughran et al., 2019). Regarding the classical cascade, immunoglobulins such as IgA, IgG, and IgM recognize and bind to specific moieties on the bacterial surface (Andre et al., 2017). Recognition is to antigens previously seen by the host during prior infection episodes or to components where naturally occurring antibody is generated, for example PC within WTA (Winkelstein and Tomasz, 1978). Regarding the alternative cascade, degradation of serum protein C3 results in the generation of C3a and C3b. C3b recognizes teichoic acid and binds to bacterial surface components initiating activation of the pathway (Hummell et al., 1981; Andre et al., 2017). The complement cascade can be blocked by serum Factor H, which the pneumococcus binds to via CbpA, which in turn binds Factor I and degrades C3b (Hyams et al., 2013). Other ways to activate complement are certain serum proteins, such as lectins, which act through the classical and lectin cascades. One of which is aforementioned CRP (Thompson et al., 1999). Many excellent reviews on the complement cascade and other serum factors that mediate host-defense are available (Ehrnthaller et al., 2011; Merle et al., 2015b; Merle et al., 2015a). 

Studies by Ren et al., have shown that PspA inhibits complement deposition on *Spn* and this feature is vital for *Spn* virulence (Ren et al., 2004). PspA, due to its being one of the most abundant CBPs, competes with CRP for the recognition of PC residues on the pneumococcal cell surface (Mukerji et al., 2012). Isogenic deficient mutants of PspA were observed to elicit greater activation of the classical complement cascade with increased deposition of Clq and C3b proteins on the bacterial surface compared to wildtype *Spn * (Li et al., 2007). Consistent with this, it has been shown by multiple investigators that PspA-deficient pneumococci are cleared more quickly from the bloodstream and are bound to a greater extent by complement, whether initiated by the alternative or classical cascade, when compared to wildtype bacteria (Tu et al., 1999; Ren et al., 2004; Mukerji et al., 2012). PspA is also able to inhibit immune adherence, where complement-bound bacteria become attached to erythrocytes and are subsequently targeted by macrophages for clearance (Li et al., 2007). By reducing the effectiveness of the complement pathways, PspA reduces the clearance of *Spn* during infection and accordingly enhances pneumococcal virulence. 

### Lactoferricin Inhibition

Human lactoferrin (hLF) is an iron-binding globular glycoprotein ~80 kDa present in mucosal secretions with a high affinity for ferric (Fe^3+^) iron (Hammerschmidt et al., 1999). Lactoferrin can be divided between an iron-bound “closed” hololactoferrin and the iron-free “open” apolactoferrin (Baker and Baker, 2005). Apolactoferrin is capable of bacteriostatic activity through iron-chelation which binds up any free exogenous iron thereby inhibiting the use of the metal by bacteria. Apolactoferrin is also capable of direct bactericidal activity *via* its breakdown and the formation of a small peptide called lactoferricin. This peptide has a large concentration of positively charged residues, similar to other cationic antimicrobial peptides, and serves to destabilize the negatively charged bacterial cell membrane (Shaper et al., 2004). Lactoferricin also interrupts the interactions between bacterial-sequestered cations, such as Ca^2+ ^and Mg^2+^, and the lipoteichoic acids in Gram-positive bacteria, leading to the de-stability and increased permeability of the cell membrane (Baker and Baker, 2005). 

The αHD of PspA contains a lactoferrin-binding region within aa residues 168-288 (Håkansson et al., 2001). This region serves to protect the bacterium from lactoferricin-mediated killing (Pérez-Dorado et al., 2012; Shaper et al., 2004). PspA showed comparably stronger affinity towards human lactoferrin compared to that from other species (Hammerschmidt et al., 1999). Håkansson et al. also reported that the ability of *Spn* to bind lactoferrin was entirely dependent on PspA. PspA versions belonging to Family 1 and Family 2 both bound to lactoferrin as demonstrated using PspA deficient pneumococci (Håkansson et al., 2001). The conservation of this trait, despite PspA’s considerable variability, highlights its importance. Accordingly, Andre et al. showed that antibodies that blocked PspA’s interaction with lactoferrin enhanced bacterial killing (André et al., 2015).

## Newly Discovered Functions of PspA

Recent publications by our group have identified new facets of *Spn* virulence involving PspA and its ability to utilize the complex host environment to its advantage. Continued elucidation of how PspA enhances pneumococcal pathogenesis will provide key information and direction towards the development of novel therapeutic or prophylactic strategies. 

### Host Lactate Utilization

During pneumococcal colonization and pneumonia, host molecules that are normally intracellular are released as a result of pneumolysin-mediated necroptosis of host cells (Labbé and Saleh, 2008; Gonzalez-Juarbe et al., 2018). These include molecules that *Spn* directly co-opts to its advantage (Van Der Sluijs et al., 2004). One newly appreciated factor is the host enzyme lactate dehydrogenase (LDH); a tetrameric protein abundant in mammalian cells composed of combinations of LDH-A and LDH-B. LDH-A converts pyruvate to lactate to generate NAD^+^ from available NADH while LDH-B acts in the reverse (Burgner and Ray, 1984). We have recently shown that *Spn* binds to host LDH-A during lung infection (**Figure 5**) (Park et al., 2021b). When mice were challenged with *Spn* that had incubated with mouse LDH-A, there was a 10-fold increase in bacterial titers collected from the lungs and an increase in bacteremia within a period of 24 hours post-infection (Park et al., 2021b). The binding site of PspA to LDH-A was determined to be the conserved 22-amino NPB that is sometimes found within the PRD (Park et al., 2021b). *Spn* that do not contain the NPB fragment within their version of PspA were unable to bind to LDH-A and did not benefit from co-incubation prior to mouse challenge. NPB is also found in some versions of CbpA and *Spn* having the NPB only in CbpA also benefitted from LDH co-incubation (Park et al., 2021b). Notably, it was determined that the enzymatic activity of LDH-A was crucial for enhancement of pneumococcal virulence. Mice challenged with *Spn* incubated with an enzymatically inactive version of LDH-A did not become hyper-virulent. Moreover, incubation of *Spn* with lactate was sufficient to confer the same effect as mixture with LDH-A. These results suggest that the NPB of PRD on PspA or CbpA co-opts host LDH-A with lactate potentially serving as a virulence-amplifying nutrient.

### Adhesion to Dying Host Cells

**Figure 3:**
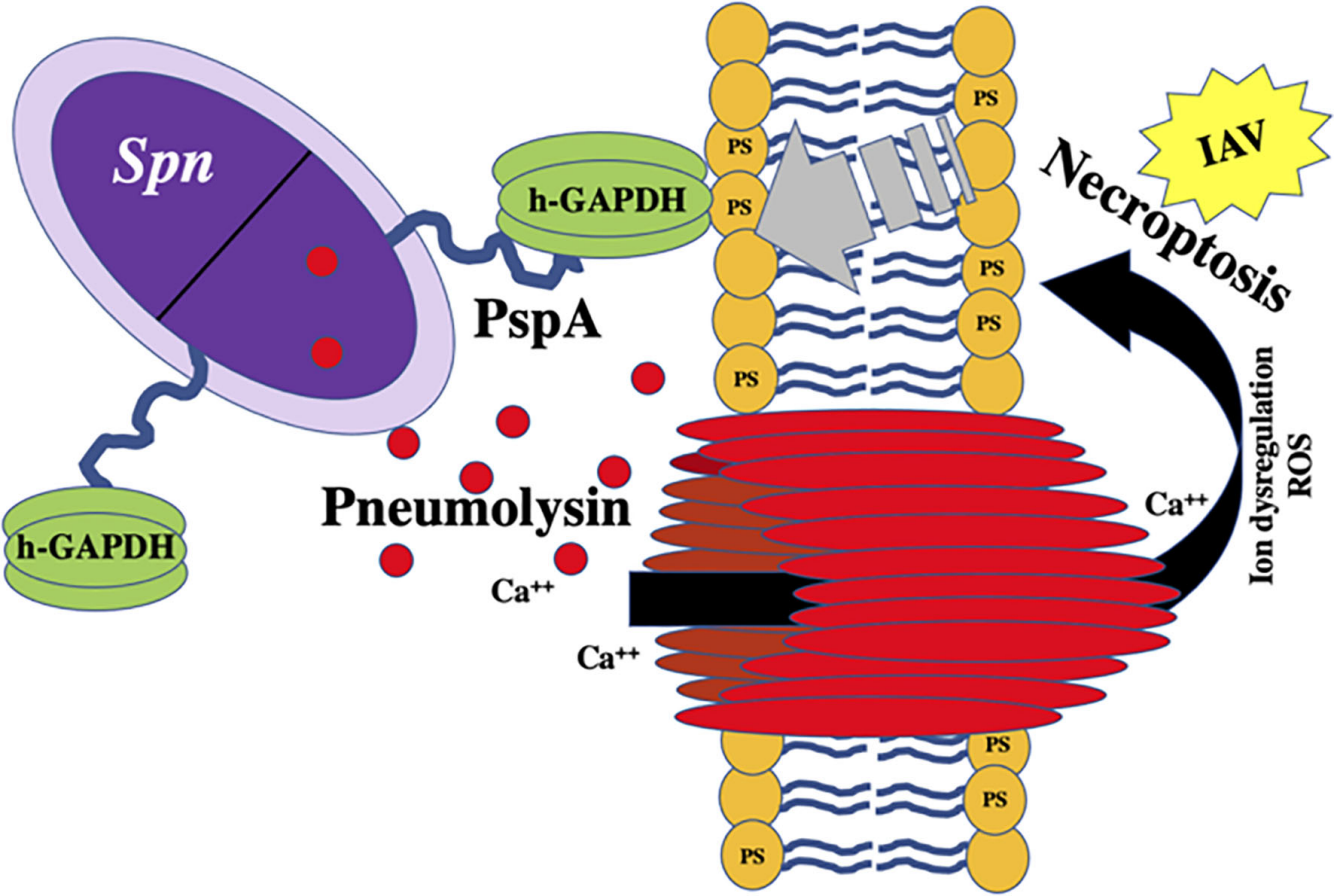
Proposed model of PspA-mediated binding to host-derived GAPDH on necroptotic cells. hGAPDH binds to dying cells *via* phosphatidylserine (PS) residues flipped from the inner to outer membrane during programmed cell death. Host cell death during *Spn* infection is primarily due to pneumolysin-mediated necroptosis. PspA binds to hGAPDH during this process. New data indicates that sensitivity of lung cells to pneumolysin-mediated necroptosis is drastically exacerbated by concomitant influenza A virus (IAV) infection (Gonzalez-Juarbe et al., 2020).

Recent findings indicate that PspA can also function as an adhesin (**Figure 3**). This property of PspA is novel as it only mediates binding to dying host cells (Park et al., 2021a). Following mammalian programmed cell death, including both necroptosis and apoptosis, the inner membrane of the host cell undergoes a process where phosphatidylserine residues are flipped outward. These residues serve as “eat me” signals to macrophages who take up the cells but also are bound by free host glyceraldehyde 3-phosphate dehydrogenase (GAPDH) (Fadok et al., 1992; Martin et al., 1995). Using pull-down assays and liquid chromatography/mass spectrometry (LC-MS), it was discovered that PspA binds to mammalian GAPDH, specifically by the amino acids 230-281 in the αHD (Park et al., 2021a). It was subsequently shown that PspA uses GAPDH to adhere to dying lung cells during infection, which impacted the bacterium’s localization within the airway thereby enhancing pneumococcal virulence during pneumonia (Park et al., 2021a). *Spn* in particular seemed to take advantage of this trait during co-infection with IAV, which sensitized cells to pneumolysin-mediated necroptosis (Gonzalez-Juarbe et al., 2020), and a condition which enhances *pspA* expression (Park et al., 2021a). It is noteworthy that the identified GAPDH-binding motif on PspA overlaps with the lactoferrin-binding domain. What is more, GAPDH binds to both apo- and holo-lactoferrin by which the host cell either egresses or acquires iron, respectively (Sheokand et al., 2014; Chauhan et al., 2015). Given the importance of lactoferrin binding, this overlap in PspA’s domains raises the possibility that *Spn* uses GAPDH as a bait to acquire iron from lactoferrin, or GAPDH may serve as a sink for lactoferrin bound to PspA thereby preventing its cleavage to lactoferricin. These vital questions require further investigation. 

### Other Interactions

In our studies that identified PspA as having affinity to lactate dehydrogenase and GAPDH, it was observed that PspA also had affinity to numerous other host proteins (Park et al., 2021a; Park et al., 2021b). Among these was the filamentous protein vimentin, which has been implicated as a key ligand for group B Streptococci and *E. coli* with regard to their ability to gain access to the central nervous system (Deng et al., 2019). Other host molecules include keratin (types I-III), pyruvate kinase, alpha-enolase, and beta-tubulin (Park et al., 2021a; Park et al., 2021b). Multiple interactions between pneumococcal surface proteins and the host is a common feature. For example, CbpA specifically binds to laminin receptor, polymeric immunoglobulin receptor, C3, and serum Factor H (Smith and Hostetter, 2000; Lu et al., 2003; Lu et al., 2006; Orihuela et al., 2009). These multiple roles reveal the efficiency of the bacterium, the highly versatile nature of the αHD of PspA and CbpA, and suggest our understanding of PspA’s interactions is most likely incomplete.

## Potential of PspA as a Vaccine Antigen 

**Figure 4:**
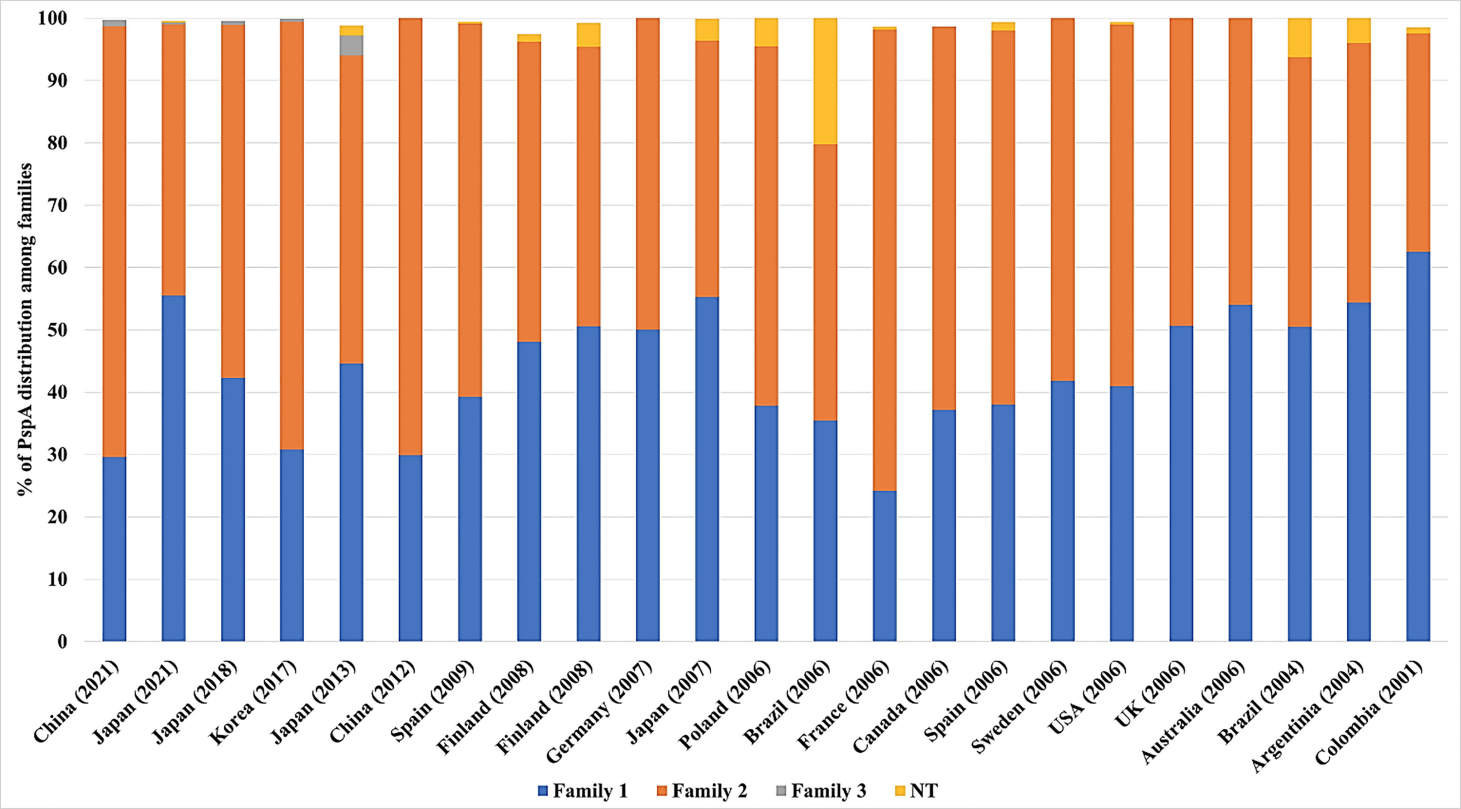
Distribution of PspA families across several countries. The proportion of PspA family 1(blue), 2 (orange), 3 (grey), and non-typable (NT-Yellow) from each study is represented as percentage (%). The origin of *Spn* and the number of isolates from each study are detailed in **Table 2** along with the corresponding reference. For some studies, not all *Spn* strains examined carried the gene for PspA. In these instances the % distribution of PspA is less than 100%.

Several epidemiological studies were conducted across different countries to evaluate the distribution of PspA families among different isolates. On average, 43.6% of isolates belonged to family 1, 53.5% of isolates belonged to family 2, 0.2% of isolates belong to family 3, and the rest are non-typable. Although there are differences in the proportions of the distribution of PspA families in different populations in the world, the vast majority are composed predominantly of families 1 and 2 (**Table 2** and **Figure 4**). Pneumococcal isolates from pediatric patients, including from middle ear fluid or nasopharyngeal secretions, reveal that the majority of PspA proteins fall into either family 2 or family 1 (Vela Coral et al., 2001; Mollerach et al., 2004; Melin et al., 2008; Jiang et al., 2021). While in adults over 50 years of age, differences in the PspA family distribution show that the majority (> 50-55%) of PspA proteins are in family 2 (Hollingshead et al., 2006). In meningitis isolates from Germany, PspA proteins were evenly distributed over families 1 and 2 with clades 1 and 3 (Heeg et al., 2007). Recent studies in Japan reported that >55% of isolates from adults over the age of 15 years old are in PspA family 1 and >55% isolates from pediatric patients under the age of 16 years are in PspA family 2 (Kawaguchiya et al., 2018; Chang et al., 2021). Additionally, epidemiological surveillance in Brazil conducted during 1977–2002 revealed 50.5% of the isolates belonged to family 1, 43.2% were members of family 2, and 6.3% were not classified. Across all studies, Family 3 was consistently the least common version of PspA. Its greatest reported prevalence came from one Japanese study that showed 3.2% of pneumococcal isolates from the upper respiratory tract belonged to Family 3 (Hotomi et al., 2013). In all, it is evident that were PspA to be used as a vaccine antigen versions must be used that provide protection against both family 1 and 2 while also considering that family 3 or other unclassifiable versions of PspA have the potential to emerge thereafter.

**Table 2:** Distribution of PspA families across different countries.

Country	Number of Isolates	Family 1	Family 2	Family 3	Non-typable	Year of Isolates	Source of Isolates	Reference
China	81	29.6	69.1	1	0	2014-2018	Pediatric patients with the median age of patients being 1.08 (0.79–3.20)	Jiang et al., 2021
Japan	1,939	55.5	43.50	0.3	0.2	2014–2019	Adult over the age of 15 years old	Chang et al., 2021
Japan	678	42.3	56.6	0.6	0	June-November 2016	Pediatric outpatients under the age of 16 years (median age 2.0 years; mean ±SD, 2.9±2.4 years)	Kawaguchiya et al., 2018
Korea	185	30.8	68.6	0.5	0	1991-2016	Children <18 years of age	Yun et al., 2017
Japan	251	44.6	49.4	3.2	1.6	January and May 2003	Upper respiratory tract infections in patients from 0 to 68 years old	Hotomi et al., 2013
China	171	29.90	70.1	0	0	2006-2008	Children <14 years of age	Qian et al., 2012
Spain	112	39.3	59.8	0	0.3	1997 -2007	Healthy children carriers and patients with invasive disease	Rolo et al., 2009
Finland	81	48.1	48.1	0	1.23	1994-1997	Nasopharyngeal carriers from children < 2 years of age	Melin et al., 2008
Finland	154	50.6	44.8	0	3.8	1994-1997	Acute otitis media from children < 2 years of age	Melin et al., 2008
Germany	40	50	50	0	0	1997-2003	Pneumococcal meningitis from children < 16 years of age	Heeg et al., 2007
Japan	141	55.3	41.1	0	3.5	2003-2004	Patients over 15 years old diagnosed with community acquired pneumonia	Ito et al., 2007
Poland	156	37.8	57.7	0	4.5	1997 -2002	Meningitis patients among different age groups	Sadowy et al., 2006
Brazil	183.0	35.5	44.3	0	20.2	2000-2001	Nasopharyngeal isolates from children < 5 years of age	Pimenta et al., 2006
France	215	24.2	74.0	0	0.5	1995-2002	Isolates from adults over 50 years of age collected in seven countries	Hollingshead et al., 2006
Canada	148	37.2	61.5	0	0.0	1995-2002	Isolates from adults over 50 years of age collected in seven countries	Hollingshead et al., 2006
Spain	150	38.0	60.0	0	1.3	1995-2002	Isolates from adults over 50 years of age collected in seven countries	Hollingshead et al., 2006
Sweden	67	41.8	58.2	0	0.0	1995-2002	Isolates from adults over 50 years of age collected in seven countries	Hollingshead et al., 2006
USA	930	41.0	58.0	0	0.4	1995-2002	Isolates from adults over 50 years of age collected in seven countries	Hollingshead et al., 2006
UK	237	50.6	49.4	0	0.0	1995-2002	Isolates from adults over 50 years of age collected in seven countries	Hollingshead et al., 2006
Australia	100	54.0	46.0	0	0.0	1995-2002	Isolates from adults over 50 years of age collected in seven countries	Hollingshead et al., 2006
Brazil	366	50.5	43.2	0	6.3	1977–2002	Epidemiological surveillance	Brandileone et al., 2004
Argentina	149	54.4	41.6	0	4.0	1993-2000	Isolates from children < 6 years of age	Mollerach et al., 2004
Colombia	40	62.5	35	0	1	1994-1998	Isolates from children <5 years of age	Vela Coral et al., 2001

The αHD of PspA has several properties that make it attractive as a vaccine protein candidate including high expression of the protein at important anatomical sites for colonization and transmission (D'Mello et al., 2020), immunogenicity and cross-reactivity of elicited antibodies (Nabors et al., 2000), accessibility for antibody binding at the cell surface (Vadesilho et al., 2014; Scott et al., 2021), and demonstrated protection in animal models of severe infection (Hollingshead et al., 2000; Miyaji et al., 2015). The PRD of PspA has also been shown to elicit an antibody response and protection against *Spn* infection (Mukerji et al., 2018). Notably, and as we continue to identify subdomains of PspA that have independent biological roles, this information can be used to select versions of PspA which are not only cross-protective as result of opsonization, but also because they are capable of neutralizing the biological activities of PspA. For example, antibodies that prevent PspA interactions with lactoferrin/GAPDH or lactate dehydrogenase, have the potential to alter bacterial resistance to host killing, its localization in the airway, and nutrient acquisition, respectively.

In addition to PspA, multiple other pneumococcal surface proteins have been studied as potential vaccine candidates (Miyaji et al., 2015; Masomian et al., 2020; Scott et al., 2021). One promising option are multivalent protein vaccines. These combine multiple antigens to increase antigen delivery and induce broader protection in the host. Candidate proteins worthy of consideration for protection against *Spn* include a pneumolysin toxoid, CbpA, and the pneumococcal histidine triad protein D (PhtD) among others (Chen et al., 2015; Scott et al., 2021). PspA in combination with pneumolysin is in particular intriguing given the importance of induced cell death on PspA binding and cell death-mediated accessibility to host-derived lactate dehydrogenase. Along such lines, it has been shown that these two proteins are synergic in regard to the protection they incur in mice when used together (Briles et al., 2000).

The major concerns regarding the use of PspA in a pneumococcal vaccine stem from the lack of cross-reactivity between all the clades. Antibody against clades 1 and 2 are cross protective, however, cross protection is reduced with consideration to clades 3, 4, and 5 (Sempere et al., 2021). This concern overlaps the mosaic nature of PspA with regard to its ability to evade the host immune response and indicates that to avoid “PspA replacement” or emergence of a non-typable PspA version, any multi-valent PspA vaccine should be developed that covers conserved regions under both positive and negative pressure by the host (Yamaguchi et al., 2019). The risk of this can also be diminished by including more than one protein in any vaccine formulation, such as pneumolysin (Sempere et al., 2021). Another concern was raised when it was found that PspA has low sequence homology with human cardiac myosin, which may elicit the production of autoantibodies against cardiac tissue leading to inflammation and tissue damage along with autoimmune disease (Ginsburg et al., 2012). To avoid this risk, antigen design can purposely exclude the region of PspA with the low myosin homology.

PspA vaccine clinical trials are ongoing. Recently, a phase I clinical trial was completed with a recombinant PspA oral vaccine developed using three different avirulent strains of *Salmonella typhi* (RASV) each expressing PspA (ClinicalTrials.gov Identifier: NCT01033409). A similar study showed promising results in mice using an oral attenuated RASV-expressing PspA vaccine (Seo et al., 2012). The future of pneumococcal vaccine design may rely less on using the capsule polysaccharides as antigenic targets and more on bacterial surface proteins such as PspA for broader and sustained protection with less risk of serotype escape. 

## Conclusion

**Figure 5:**
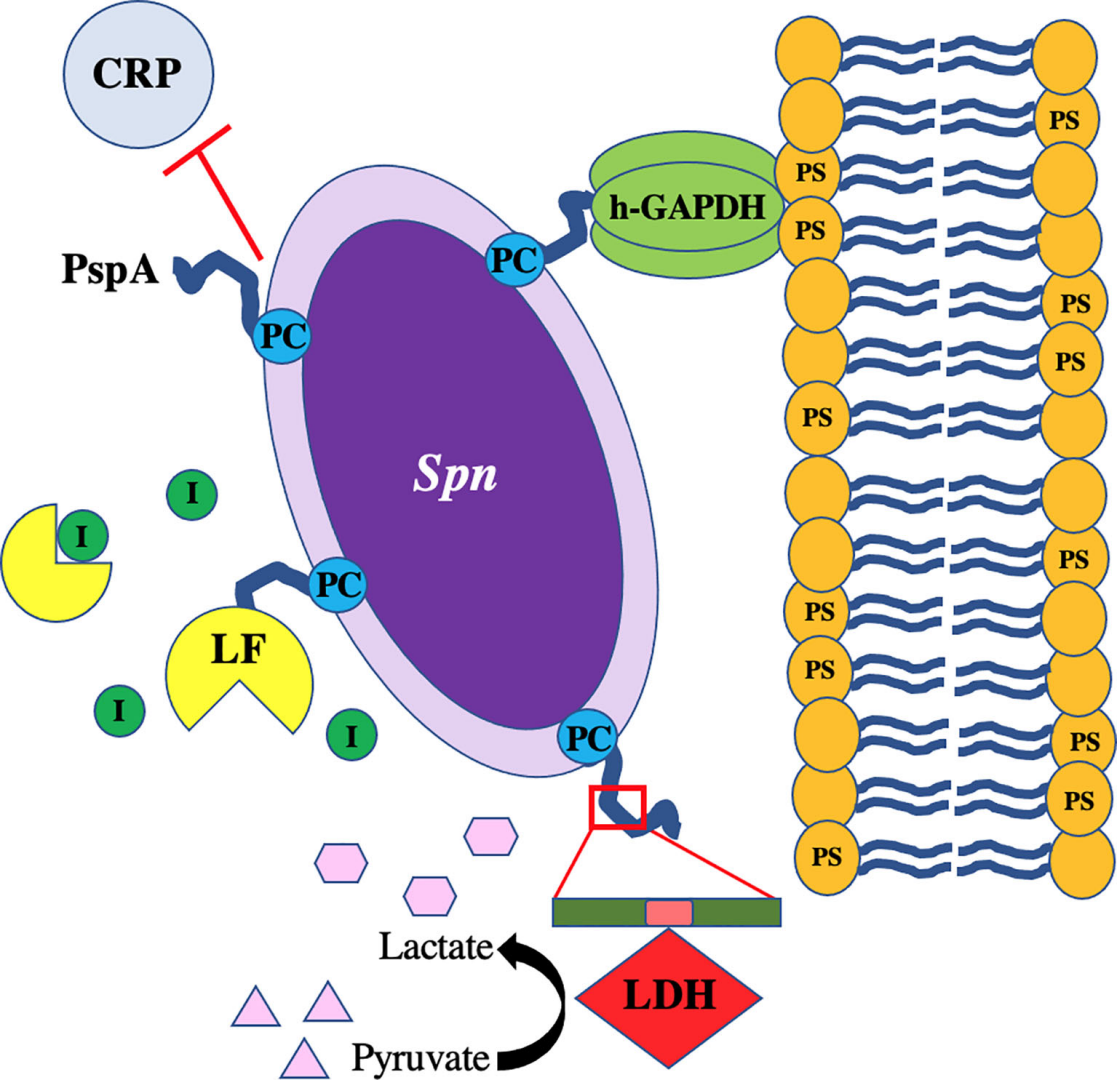
PspA interacts with *Streptococcus pneumoniae* as a “Jack of All Trades.” The pneumococcus with PspA (in blue) extending beyond the capsule which blocks the binding of C-reactive protein (CRP) to phosphorylcholine (PC) on the bacterial surface and acts as an adhesin by binding to host GAPDH. *Spn* co-opts host lactate dehydrogenase (LDH) bound to the non-proline block (in pink) of the proline-rich domain (in green) on PspA and uses converted lactate as a nutrient in poor environmental conditions. PspA on the surface of the pneumococcus also binds to lactoferrin (LF) to protect the bacterium from killing by lactoferricin.

*Spn* continues to be a leading cause of respiratory diseases such as community-acquired pneumonia with transmission rates high amongst substantial portions of the population. Current treatment options against pneumococcal infections rely on antibiotics, which are dwindling due to the spread of resistance, whereas prophylactic options are centered on the polysaccharide-based vaccines, with serotype escape beginning to impact overall efficacy. *Spn* has virulence factors that allow it to invade, colonize, and infect its host and these have been studied extensively over the years. Surface proteins on *Spn* such as PspA have garnered renewed interest as potential vaccine candidates due to its presence on nearly all clinical isolates along with high expression across different anatomical sites in the host. Reports have shown promising results in animal models of *Spn* infection where immunization against PspA protects against severe disease and enhances clearance of the bacteria *via* opsonophagocytosis. Vaccine designs using PspA will have to consider low homology with cardiac myosin to avoid undue inflammation or activation of autoantibodies. Other CBPs are also in consideration, highlighting the move away from dependence on capsule-based antibody recognition by the host and more towards protein-based formulations. New information regarding PspA virulence and its ability to act as a “Jack of all Trades” have improved our understanding of this complex protein and help guide future research questions (**Figure 5**). As such, continued studies into the molecular mechanisms of PspA during pneumococcal dissemination and infection will be necessary to fully elucidate the vital role this virulence factor plays in *Spn* pathogenesis and possible targeting for development of novel therapies. 

## Author Contributions

JL wrote the first draft of the paper. JL, MT, DB and CO contributed to the subsequent writing, editing, and direction of the paper. All authors approved the submitted version.

## Funding

CO receives support from NIH grants AI156898, AI148368, AI114800, and AI146149.

## Conflict of Interest

The authors declare that the research was conducted in the absence of any commercial or financial relationships that could be construed as a potential conflict of interest.

## Publisher’s Note

All claims expressed in this article are solely those of the authors and do not necessarily represent those of their affiliated organizations, or those of the publisher, the editors and the reviewers. Any product that may be evaluated in this article, or claim that may be made by its manufacturer, is not guaranteed or endorsed by the publisher.
